# Tissue and Isoform-Specific Effects of Platelet-Derived Growth Factor on Neonatal-Derived Dermal and Fetal-Derived Lung Fibroblast Profibrotic Functions

**DOI:** 10.3390/cells15070637

**Published:** 2026-04-01

**Authors:** Brandon Kohlen, Raveen Badyal, Kevin J. Keen, James V. Dunne, Tillie-Louise Hackett

**Affiliations:** 1 Centre for Heart Lung Innovation, St. Paul’s Hospital, Vancouver, BC V6Z 1Y6, Canada; raveen.badyal@hli.ubc.ca (R.B.); kevin.keen@hli.ubc.ca (K.J.K.); james.dunne@vch.ca (J.V.D.); tillie.hackett@hli.ubc.ca (T.-L.H.); 2 Department of Anesthesiology, Pharmacology and Therapeutics, The University of British Columbia, Vancouver, BC V6T 1Z3, Canada; 3 Department of Mathematics and Statistics, The University of Northern British Columbia, Prince George, BC V2N 4Z9, Canada; 4 Department of Medicine, The University of British Columbia, Vancouver, BC V6T 1Z3, Canada

**Keywords:** fibroblasts, fibrosis, platelet-derived growth factor, extracellular matrix, systemic sclerosis, idiopathic pulmonary fibrosis, inflammation, collagen contraction, proliferation

## Abstract

**Highlights:**

**What are the main findings?**

- PDGF isoforms induce distinct profibrotic profiles in lung and dermal fibroblasts that underscore the need for tissue-specific treatment strategies for fibrosis.
- In dermal fibroblasts, PDGF-BB is the predominant PDGF isoform that induces fibroblast contraction, proliferation, and IL-11 and IL-6-mediated signalling due to elevated PDGF Receptor-β expression and ERK1/2 signalling, compared to lung fibroblasts.

**What are the implications of the main findings?**

- PDGF is important for driving dermal fibroblast proliferation, ECM contraction and profibrotic cytokine release, but not ECM synthesis or myofibroblast differentiation.
- Isoform-specific PDGF therapeutics inhibiting PDGF-BB may offer antifibrotic benefit with reduced side effects in dermal fibrotic diseases such as systemic sclerosis.

**Abstract:**

Elevated levels of platelet-derived growth factor (PDGF) isoforms in fibrosis are implicated in driving a dysfunctional profibrotic fibroblast phenotype. This study investigated the differential effects of the five PDGF isoforms (AA, AB, BB, CC, and DD) in inducing neonatal dermal and fetal lung fibroblast contraction, proliferation, cytokine production, myofibroblast differentiation, and extracellular matrix (ECM) deposition. All PDGF isoforms, except PDGF-AA, increased contraction of 3-dimensional collagen I gels by dermal (*p* < 0.01) and lung fibroblasts (*p* < 0.05) compared to media control. PDGF-AB, BB, and CC enhanced proliferation only in dermal fibroblasts (*p* < 0.05). PDGF-BB induced profibrotic IL-11 cytokine release in dermal and lung fibroblasts (*p* < 0.0001) and IL-6 cytokine release in dermal fibroblasts (*p* < 0.05) compared to media control. None of the PDGF isoforms affected ECM synthesis or myofibroblast differentiation. Dermal fibroblasts exhibited elevated PDGF Receptor-β (PDGFRβ) expression (*p* < 0.01) and increased basal ERK1/2 phosphorylation (*p* < 0.05) compared to lung fibroblasts. In summary, PDGF modulates fibroblast functions in a tissue-specific manner, with PDGF-BB driving profibrotic processes in dermal fibroblasts through high PDGFRβ expression and ERK1/2 signalling. Further research is needed to explore the benefit of tissue and isoform-specific PDGF inhibition strategies in skin and lung fibrosis.

## 1. Introduction

Fibroblasts are central to tissue homeostasis and repair, orchestrating the synthesis, remodelling, and spatial organization of the extracellular matrix (ECM) [1]. The ECM not only provides a structural scaffold, but also stores bioactive macromolecules and mediators that regulate cellular behaviour through biochemical and biomechanical cues. During normal repair, fibroblast activity is tightly regulated by growth factors and cytokines released from the surrounding epithelium, stroma, and immune cells in response to pathogen exposure, tissue injury, and cellular stress [2,3]. Dysregulation of these signalling cues leads to excessive ECM deposition and persistent fibroblast activation, resulting in fibrotic tissue that is structurally, abnormally, and functionally impaired [4].

Among the growth factors that influence fibroblast function, the platelet-derived growth factor (PDGF) signalling axis is a potent driver of proliferation, migration, survival, and ECM production in dermal and lung fibroblasts [5,6,7,8]. PDGF signalling is also essential for the development and maintenance of multiple organs, including the lung, skin, kidney, and cardiovascular system [9]. Elevated levels of PDGF have been reported in fibrotic diseases such as systemic sclerosis (SSc) and idiopathic pulmonary fibrosis (IPF), and PDGF-mediated signalling is thought to promote profibrotic fibroblast activation and altered ECM deposition [6,10]. IPF is the most common form of interstitial lung disease (ILD) resulting in lung fibrosis and median survival from diagnosis of 3–5 years [11,12]. Similarly, SSc is characterized by skin fibrosis and chronic immune activation that can progress to ILD, which is a major determinant of morbidity and mortality for patients with SSc [13,14]. In SSc and IPF, aberrant ECM composition and increased circulating growth factors, including PDGF, are thought to maintain fibroblasts in a chronic activated myofibroblast-like state, leading to skin and lung fibrosis [5,15,16]. Despite its recognized importance, the isoform-specific actions of PDGF in fibroblast-driven fibrosis in the skin and lung remain poorly understood.

The PDGF family consists of four subunits (PDGF-A, B, C, and D) that dimerize into four homodimers (PDGF-AA, BB, CC, and DD) and one heterodimer (PDGF-AB), which signal through dimeric PDGF receptors (PDGFRαα, αβ, and ββ) [17]. It is understood that PDGF-A, B, and C are capable of binding to the PDGFRα chain while PDGF-B and PDGF-D bind to the PDGFRβ chain [9,17,18]. Previous studies have typically focused on one or two PDGF isoforms, predominantly PDGF-AA or PDGF-BB, leaving significant knowledge gaps in PDGF isoform-specific effects. The present study comprehensively investigates the functional effects of all five PDGF isoforms on neonatal-derived dermal and fetal-derived lung fibroblasts, which are commercially available human, non-transformed cell lines, thereby enabling independent studies to reproduce the results. The goal was to define isoform-specific PDGF pathways for further investigation in primary adult fibroblasts that may be selective in limiting skin and lung fibrosis without impairing tissue repair.

## 2. Materials and Methods

### 2.1. Cells

The human neonatal skin fibroblast cell line (BJ, cat. CRL-2522) and human fetal lung fibroblast cell line (HFL1, cat. CCL-153) were purchased from American Type Culture Collection (ATCC, Manassas, VA, USA). Fibroblasts were seeded at 80,000 cells per well in 6-well plates and grown in 10% fetal bovine serum (FBS, cat. 12483020, Thermo Fisher Scientific, Waltham, MA, USA) with 1% penicillin–streptomycin fungizone (PSF, cat. SV3007901, Cytiva, Marlborough, MA, USA) in Dulbecco’s Modified Eagle Medium (DMEM, cat. 11965126, Thermo Fisher Scientific) at 37 °C with 5% CO_2_ until 80% confluency. Fibroblasts were then serum-starved with DMEM containing 1% FBS for 16 h. Following a dose–response study (Supplemental Figure S1) and review of prior publications, fibroblasts were treated with control media or 25 ng/mL of human recombinant PDGF isoforms PDGF-AA (cat. 78095), PDGF-AB (cat. 78096), PDGF-BB (cat. 78097), PDGF-CC (cat. 78168), or PDGF-DD (cat. 78222), from STEMCELL Technologies (Vancouver, BC, Canada), for 72 h. The experiments were approved by the Providence Health Care Research Ethics Board (H13-02173) at the University of British Columbia.

### 2.2. Proliferation Assay

After a 72-h treatment with the PDGF isoforms and control media, cells were dissociated using 0.25% Trypsin-EDTA (cat. 25200072, Thermo Fisher Scientific), and the live cell number was quantified with the Countess II (Thermo Fisher Scientific). Cells were also treated with 10 ng/mL of human recombinant transforming growth factor (TGF)-β1 (cat. 100-21-10UG, Thermo Fisher Scientific) for comparison with a profibrotic growth factor known to be upregulated in fibrosis.

### 2.3. Contraction Assay

A 3-dimensional (3D) collagen I gel contraction assay was used as previously described [19]. Briefly, bovine serum albumin (BSA, cat. A9418) was sourced from Millipore Sigma (Oakville, ON, Canada) and used to coat each well of a 12-well plate with a 1% BSA in DMEM solution for 2 h. Collagen I, derived from rat tail (cat. CACB354236, Corning, Corning, NY, USA), was diluted to 0.4 mg/mL in DMEM and 1 mL was dispensed into each well. After a 16-h incubation period at 37 °C with 5% CO_2_, a pipette tip was used to separate the gel from the wall of each well. Gels were treated with control media or the five PDGF isoforms at 25 ng/mL, and then fibroblasts were seeded at 40,000 cells/well. As fibroblasts contract the collagen gel, media is extruded from the gel, decreasing its weight. Contracted gels were weighed on an analytical balance to assess the percentage of contraction by normalizing to the uncontracted gel weights as previously described [19].

### 2.4. Enzyme-Linked Immunosorbent Assay (ELISA)

Cell-free supernatant was used to assess the release of IL-6 (cat. DY206-05), IL-8 (cat. DY208), and IL-11 (cat. DY218) using DuoSet ELISAs, manufactured by R&D Systems (Minneapolis, MN, USA). Absorbance was read at 450 nm with a 570 nm reference wavelength on the SpectraMax iD3 (Molecular Devices, San Jose, CA, USA).

### 2.5. Western Blot

Fibroblast protein lysates were extracted using cell extraction buffer (cat. FNN0011, Thermo Fisher Scientific) containing phosphatase inhibitor (cat. P5726-5ML, Millipore Sigma), protease cocktail inhibitor (cat. P2714-1BTL, Millipore Sigma), and phenylmethylsulfonyl fluoride (cat. 10837091001, Millipore Sigma). Samples were combined with loading dye (cat. 928-40004, LI-COR Biotech, Lincoln, NE, USA) and heated at 95 °C for 7 min. Protein concentration was assessed with a Pierce BCA Assay (cat. 22662, Thermo Fisher Scientific). Approximately 7.5 µg of protein was loaded per well in a 4–20% Mini-PROTEAN TGX Precast Protein Gel (cat. 4561096, Bio-Rad, Hercules, CA, USA). Protein was transferred to a 0.45 μm nitrocellulose membrane (cat. 1620115, Bio-Rad) and total protein was quantified with the Revert 700 Total Protein Stain Kit (cat. 926-11016, LI-COR Biotech), following the manufacturer’s instructions. Membranes were blocked for one hour at room temperature with Intercept Phosphate-Buffered Saline (PBS) Blocking Buffer (cat. 927-70001, LI-COR Biotech) and probed overnight at 4 °C with anti-collagen-I antibody (1:1000 dilution, clone EPR7785, cat. ab138492, Abcam, Waltham, MA, USA), anti-fibronectin antibody (1:2000 dilution, clone IST-9, cat. sc-59826, Santa Cruz Biotechnology, Dallas, TX, USA), anti-alpha-smooth muscle actin polyclonal antibody (1:300 dilution, cat. PA5-85070, Thermo Fisher Scientific), anti-PDGFR alpha antibody (1:1000 dilution, clone EPR22059-270, cat. ab203491, Abcam), anti-PDGFR beta antibody (1:1000 dilution, clone 42G12, cat. ab69506, Abcam), anti-ERK1/ERK2 antibody (1:500 dilution, clone ERK-7D8, cat. 13-6200, Thermo Fisher Scientific), or anti-phospho-ERK1/2 antibody (1:1000 dilution, clone 2P8Q2, cat. MA5-38228, Thermo Fisher Scientific). Antibody dilutions were prepared in Intercept PBS Blocking Buffer with 0.2% Tween 20 (cat. P9416, Millipore Sigma). Membranes were washed twice for five minutes with PBS plus 0.1% Tween 20 and then once for 10 min with PBS. Membranes were probed with the appropriate secondary antibody (1:5000 dilution, cat. 926-32212, 926-32211, 926-68071, LI-COR Biotech) at room temperature for one hour. The wash steps were repeated before imaging the membranes with the LI-COR Odyssey CLx. Image Studio Lite version 5.2 (LI-COR Biotech) software was used to quantify protein expression.

### 2.6. Lactate Dehydrogenase (LDH) Cytotoxicity Assay

Cell death was quantified using the LDH assay kit (cat. ab65393) with a standard curve of recombinant human lactate dehydrogenase protein (cat. ab93699), both sourced from Abcam. Samples were loaded in duplicate with 10 µL/well and cytotoxicity was calculated following the manufacturer’s instructions.

### 2.7. Statistical Analysis

The data were assessed for outliers using the ROUT (robust regression followed by outlier identification) method [20]. Comparisons between PDGF isoforms were performed using a one-way analysis of variance (ANOVA) and corrected for multiple comparisons using a Dunnett’s test in GraphPad Prism 10. A linear mixed-effects model was used to assess the interaction effect between PDGF and TGF-β1 on IL-6 and IL-11 secretion. A *p*-value < 0.05 was considered significant.

## 3. Results

### 3.1. PDGF Isoforms AB, BB, CC, and DD Are Potent Inducers of Dermal and Lung Fibroblast Collagen Contraction

**Figure 1:**
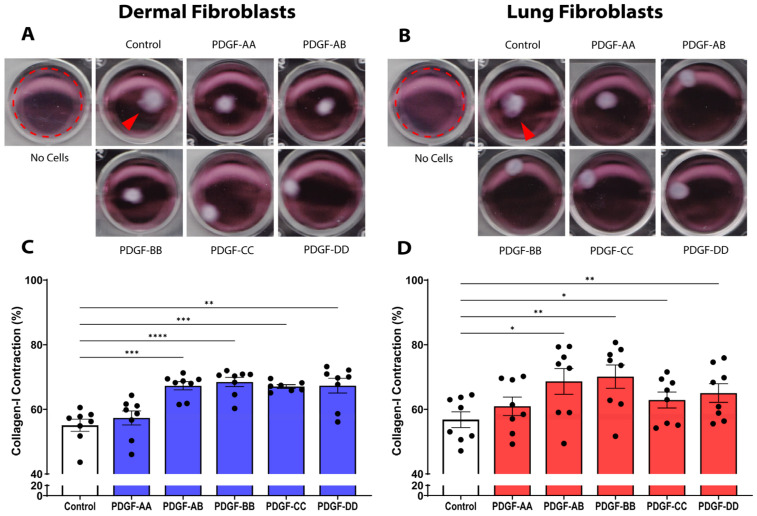
PDGF stimulates the contraction of 3D collagen I gels. Representative images of dermal (**A**) and lung fibroblasts (**B**) seeded at a density of 40,000 cells/well in a 0.4 mg/mL collagen I gel and incubated for 72 h with 25 ng/mL PDGF-AA, AB, BB, CC, DD, or media control. The collagen gel with no fibroblasts demonstrates no contraction. The dotted red circle highlights the uncontracted collagen gel with red arrows in the control wells representing contracted collagen gels. Graphs show the percentage of collagen I contraction in dermal ((**C**), *n* = 8 biological replicates) and lung fibroblasts ((**D**), *n* = 8 biological replicates). Data represent the mean with standard error of the mean (SEM) and each black dot represents a biological replicate. A one-way ANOVA was used to assess differences between conditions compared to control. * *p* < 0.05, ** *p* < 0.01, *** *p* < 0.001, **** *p* < 0.0001.

Contraction of fibrillar collagen fibres during wound repair is an important fibroblast response for wound closure, and the increase in contraction of collagen fibres leads to increased tissue density during fibrosis [16,21]. As shown by the representative images in Figure 1A,B, we assessed the ability of the five PDGF isoforms to promote dermal and lung fibroblast contraction of a 3-dimensional 0.4 mg/mL collagen I gel. In dermal fibroblasts, the PDGF isoforms AB (*p* < 0.001), BB (*p* < 0.0001), CC (*p* < 0.001), and DD (*p* < 0.01) enhanced contraction of the collagen I gel, compared to the media control (Figure 1C). In lung fibroblasts, the PDGF isoforms AB (*p* < 0.05), BB (*p* < 0.01), CC (*p* < 0.05), and DD (*p* < 0.01) enhanced collagen I contraction, compared to the media control (Figure 1D). In summary, PDGF-AB, BB, CC, and DD can increase collagen I contraction and remodelling in dermal and lung fibroblasts, leading to increased tissue density and fibrosis.

### 3.2. PDGF Isoforms AB, BB, and CC Stimulate Dermal Fibroblast Proliferation

**Figure 2:**
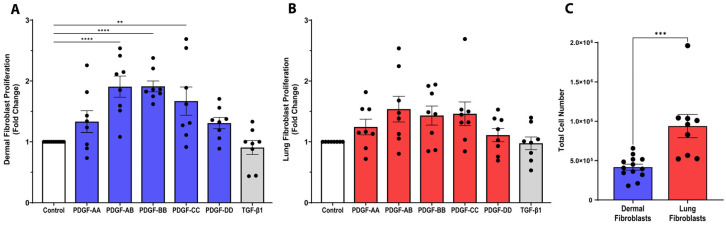
PDGF enhances proliferation of dermal fibroblasts. Dermal and lung fibroblasts were seeded at a density of 80,000 cells/well. Fibroblasts were stimulated with 25 ng/mL PDGF-AA, AB, BB, CC, or DD, or 10 ng/mL TGF-β1 for 72 h, and the fold change in cell number is shown for dermal ((**A**), *n* = 8 biological replicates) and lung ((**B**), *n* = 8 biological replicates) fibroblasts. The baseline comparison of proliferation is shown for dermal and lung fibroblasts (**C**) as total cell number. Data represent the mean with SEM and each black dot represents a biological replicate. A one-way ANOVA was used to assess differences between conditions compared to control for fold change in proliferation and a *t*-test was used to assess differences between total cell number. ** *p* < 0.01, *** *p* < 0.001, **** *p* < 0.0001.

In fibrotic tissues, the number of fibroblasts is known to increase [22]. We next investigated the potential for the PDGF isoforms to enhance dermal and lung fibroblast proliferation. As shown in Figure 2A, PDGF-AB and BB isoforms induced a 1.9-fold increase (*p* < 0.0001) and PDGF-CC stimulated a 1.7-fold increase (*p* < 0.01) in the proliferation of dermal fibroblasts over 72 h, compared to control media. 

In lung fibroblasts, when correcting for multiple comparisons, none of the PDGF isoforms enhanced proliferation (Figure 2B); however, when not correcting using Fisher’s least significant difference (LSD) test, the PDGF isoforms AB, BB, and CC did significantly induce lung fibroblast proliferation (Supplemental Figure S2). Further, lung fibroblasts, in general, were more proliferative at baseline compared to dermal fibroblasts (Figure 2C, *p* < 0.001). We also assessed the effect of TGF-β1, which has previously been shown to modulate fibroblast proliferation, but it did not significantly change proliferation in either dermal or lung fibroblast cell lines [23,24]. Using a cytotoxicity assay, we confirmed that PDGF isoform stimulation did not induce cell death in dermal or lung fibroblasts (Supplemental Figure S3). In summary, PDGF-AB, BB, and CC induce proliferation in dermal fibroblasts, but not lung fibroblasts.

### 3.3. PDGF Isoforms Do Not Induce Myofibroblast α-Smooth Muscle Actin Expression

**Figure 3:**
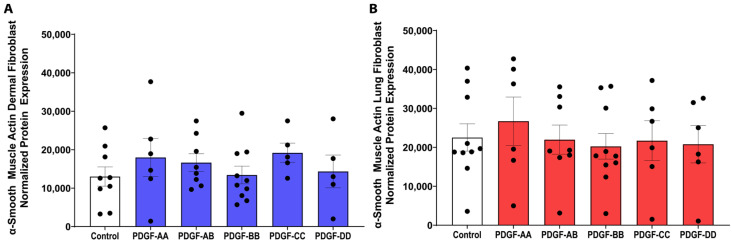
PDGF does not induce myofibroblast α-smooth muscle actin (α-SMA) expression. Fibroblasts were seeded at a density of 80,000 cells/well in a 6-well plate and stimulated with 25 ng/mL PDGF-AA, AB, BB, CC, or DD for 72 h. Protein lysate was assessed by Western blotting for protein expression of α-SMA from dermal ((**A**), *n* = 5–10 biological replicates) and lung ((**B**), *n* = 6–10 biological replicates) fibroblasts. Relative expression was normalized with a total protein stain as per the manufacturer’s instructions. A one-way ANOVA was used to assess differences between conditions compared to control. Data represent the mean with SEM and each black dot represents a biological replicate.

Increased fibroblast-to-myofibroblast differentiation has been observed in fibrosis, and these cells are characterized by increased α-smooth muscle actin (α-SMA) expression [16]. We found that none of the PDGF isoforms modified the protein expression of α-SMA, compared to the control media (Figure 3), indicating that PDGF does not stimulate dermal and lung fibroblasts to differentiate into myofibroblasts.

### 3.4. PDGF Is a Potent Inducer of Profibrotic IL-11 Cytokine Release

**Figure 4:**
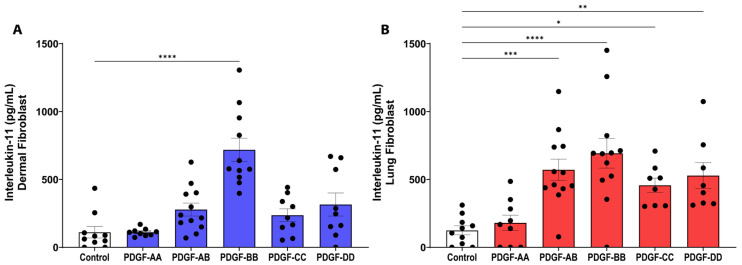
PDGF can promote interleukin (IL)-11 signalling. Fibroblasts were seeded at a density of 80,000 cells/well in a 6-well plate, stimulated with 25 ng/mL PDGF-AA, AB, BB, CC, or DD for 72 h, then assessed for IL-11 release using ELISA as shown for dermal ((**A**), *n* = 10–12 biological replicates) and lung ((**B**), *n* = 8–12 biological replicates) fibroblasts. Data represent the mean with SEM and each black dot represents a biological replicate. A one-way ANOVA was used to assess differences between conditions compared to control. * *p* < 0.05, ** *p* < 0.01, *** *p* < 0.001, **** *p* < 0.0001.

IL-11 is a profibrotic cytokine that can induce TGF-β-mediated fibrosis in animal models [25,26,27]. PDGF-BB was the only isoform to stimulate IL-11 release from dermal fibroblasts (Figure 4A, *p* < 0.0001). In lung fibroblasts, the PDGF isoforms AB, BB, CC, and DD all stimulated IL-11 release (*p* < 0.05), with PDGF-BB stimulating the strongest response (*p* < 0.0001). Therefore, PDGF may play an important role in driving fibrotic processes through IL-11 signalling, particularly the PDGF-BB isoform.

### 3.5. PDGF-BB Stimulates the Release of IL-6 from Dermal Fibroblasts

**Figure 5:**
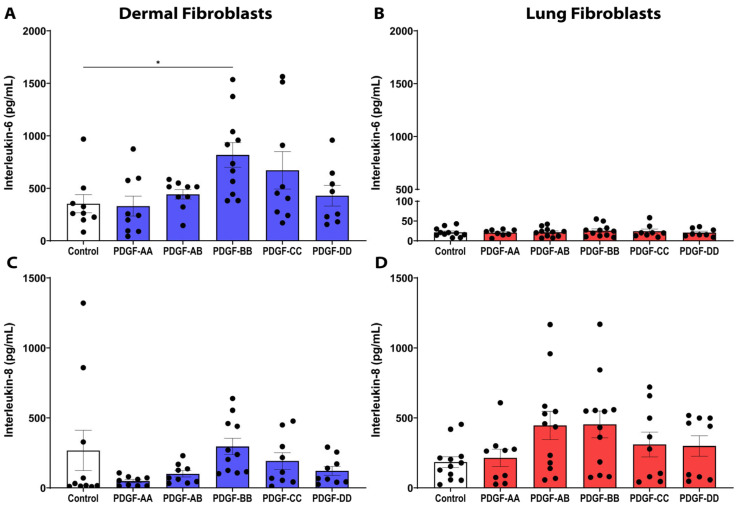
PDGF-BB stimulates proinflammatory interleukin (IL)-6 release from dermal fibroblasts. Fibroblasts were seeded at a density of 80,000 cells/well in a 6-well plate and stimulated with 25 ng/mL PDGF-AA, AB, BB, CC, or DD, and supernatant was assessed for IL-6 and IL-8 release with an ELISA after a 72-h treatment duration. Graphs show *n* = 8–12 biological replicates for the amount of IL-6 release for dermal (**A**) and lung (**B**) fibroblasts and the amount of IL-8 release for dermal (**C**) and lung (**D**) fibroblasts. Data represent the mean with SEM and each black dot represents a biological replicate. A one-way ANOVA was used to assess differences between groups compared to control. * *p* < 0.05.

IL-6 is upregulated in many fibrotic diseases, including SSc and IPF, and has been shown to promote collagen production and enhance TGF-β-driven fibrosis [28,29]. In dermal fibroblasts, PDGF-BB was able to induce a significant increase in the release of IL-6 compared to control media (Figure 5A, *p* < 0.05). However, in lung fibroblasts, none of the PDGF isoforms stimulated IL-6 release (Figure 5B). We also assessed the expression of IL-8, an acute-phase marker of inflammation and neutrophil chemoattractant, but PDGF did not induce a significant release of IL-8 in either dermal (Figure 5C) or lung (Figure 5D) fibroblasts.

### 3.6. PDGF and TGF-β1 Co-Stimulation Is Not Synergistic

**Figure 6:**
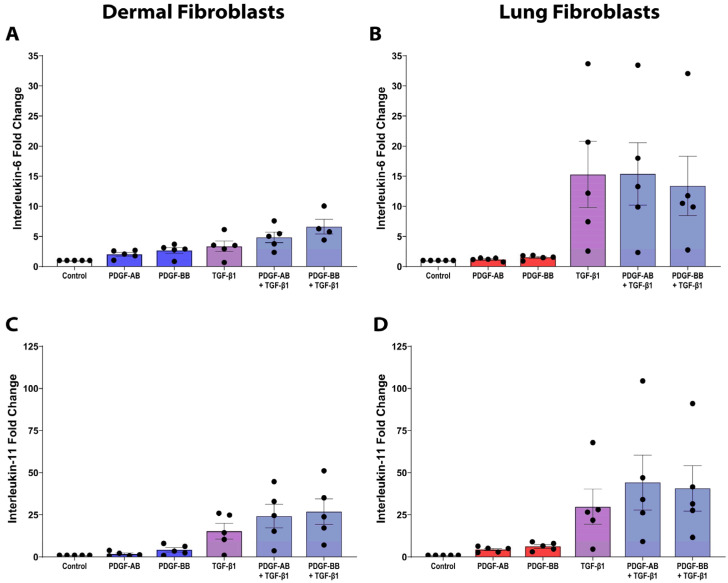
PDGF and TGF-β1 are not synergistic. Fibroblasts were seeded at a density of 80,000 cells/well in a 6-well plate and stimulated with 10 ng/mL TGF-β1 alone or in combination with 25 ng/mL PDGF-AB or PDGF-BB. Supernatant was assessed for IL-6 and IL-11 release with an ELISA after a 72-h treatment duration. Graphs show fold change relative to control media of *n* = 5 biological replicates for IL-6 release from dermal (**A**) and lung (**B**) fibroblasts and IL-11 release from dermal (**C**) and lung (**D**) fibroblasts. Data represent the mean with SEM and each black dot represents a biological replicate. A linear mixed-effects model was used to assess the interaction effect between PDGF and TGF-β1, resulting in PDGF-BB and TGF-β1 co-stimulation inducing a significant negative synergistic interaction only for lung fibroblast IL-11 release (*p* = 0.033).

To assess the proinflammatory and profibrotic cytokine responses of dermal and lung fibroblasts under fibrotic conditions, the effects of PDGF and TGF-β1 following combined stimulation were examined for IL-6 (Figure 6A,B) and IL-11 expression (Figure 6C,D). Using a linear mixed-effects model, we tested for an interaction between PDGF and TGF-β1. Across all treatment conditions in dermal and lung fibroblasts, combined PDGF-BB and TGF-β1 stimulation resulted in a significant negative interaction only for lung fibroblast IL-11 release (*p* = 0.033), consistent with an antagonistic (negative synergistic) effect.

### 3.7. PDGF Does Not Induce ECM Synthesis in Dermal or Lung Fibroblasts

**Figure 7:**
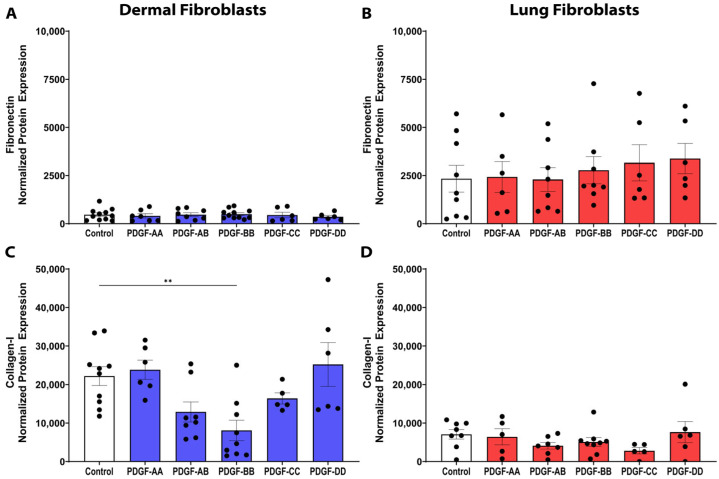
PDGF-BB inhibits collagen I protein expression from dermal fibroblasts. Fibroblasts were seeded at a density of 80,000 cells/well in a 6-well plate and stimulated with 25 ng/mL PDGF-AA, AB, BB, CC, or DD. Fibroblast protein lysate was isolated from *n* = 6–12 biological replicates and assessed with Western blotting for protein expression of fibronectin from dermal (**A**) and lung (**B**) fibroblasts and collagen I from dermal (**C**) and lung (**D**) fibroblasts. Relative expression was normalized with a total protein stain. Data represent the mean with SEM and each black dot represents a biological replicate. A one-way ANOVA was used to assess differences between conditions compared to control. ** *p* < 0.01.

Given that fibrosis is characterized by excessive ECM deposition, we next examined the synthesis of collagen I, the most prominent ECM component in fibrotic tissue, and fibronectin, a key glycoprotein facilitating fibroblast–ECM interactions [28]. The PDGF isoforms had no effect on fibronectin expression in either dermal or lung fibroblasts (Figure 7A,B). Stimulation with the PDGF isoforms did not induce collagen I protein expression; notably, PDGF-BB inhibited collagen I synthesis by dermal fibroblasts (Figure 7C, *p* < 0.01). In lung fibroblasts, the PDGF isoforms did not modulate collagen I protein expression (Figure 7D). The reduction in collagen I expression observed in PDGF-BB-treated dermal fibroblasts suggests that PDGF signalling may exert context-dependent antifibrotic effects in the dermis.

### 3.8. PDGF Receptor-β Is Expressed over Two-Fold Higher than PDGF Receptor-α in Dermal Fibroblasts

**Figure 8:**
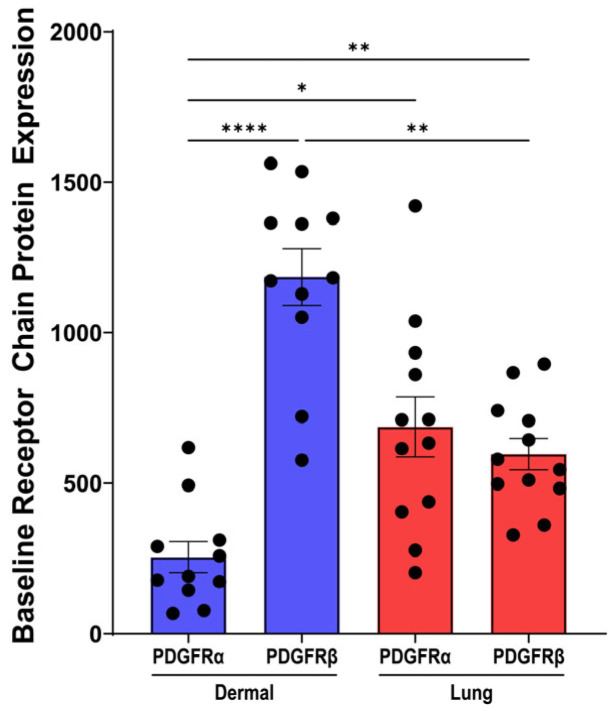
The heterogeneity of PDGF receptor expression in dermal and lung fibroblasts. Fibroblasts were seeded at a density of 80,000 cells/well in a 6-well plate and treated with control media for 72 h. Protein lysate was isolated from fibroblasts and assessed with Western blotting for baseline protein expression of PDGFRα and PDGFRβ from dermal and lung fibroblasts (*n* = 12 biological replicates). Relative expression was normalized with a total protein stain. Data represent the mean with SEM and each black dot represents a biological replicate. A one-way ANOVA with Tukey’s multiple comparisons test was used to assess differences in receptor expression between dermal and lung fibroblasts. * *p* < 0.05, ** *p* < 0.01, **** *p* < 0.0001.

To determine a potential explanation for the differential effects of the PDGF isoforms in dermal and lung fibroblasts, the expression of the PDGF receptor α chain (PDGFRα) and β chain (PDGFRβ) was assessed. At baseline, dermal fibroblasts expressed significantly greater expression of the PDGFRβ chain compared to the PDGFRα chain (Figure 8, *p* < 0.0001). The protein expression of the PDGFRα and PDGFRβ chains in lung fibroblasts was comparable. Comparing the protein expression of PDGFRβ between the two fibroblast tissue types demonstrated that PDGFRβ has greater expression in dermal fibroblasts compared to lung fibroblasts (Figure 8, *p* < 0.01). In dermal fibroblasts, greater PDGFRβ protein expression may suggest why the PDGF-AB and BB isoforms had a greater effect on cytokine release and proliferation compared to lung fibroblasts with lower PDGFRβ expression.

### 3.9. PDGF Treatment Does Not Modulate PDGF Receptor Expression Compared to Control

**Figure 9:**
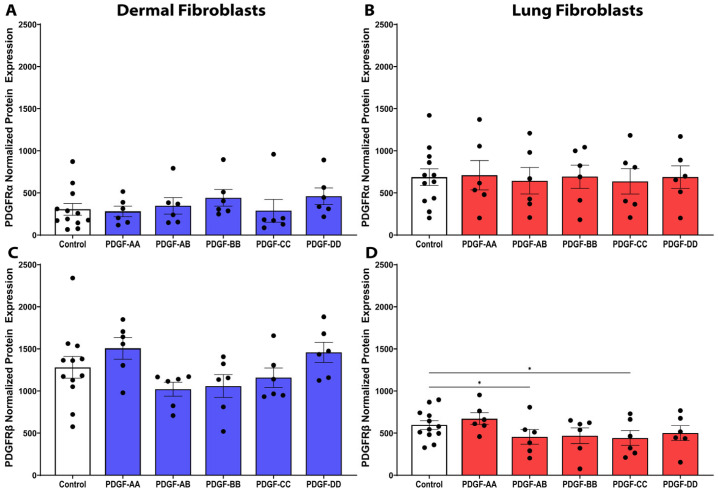
PDGF receptor expression remains largely unchanged with PDGF stimulation. Fibroblasts were seeded at a density of 80,000 cells/well in a 6-well plate and stimulated with 25 ng/mL PDGF-AA, AB, BB, CC, or DD. Fibroblast protein lysate was isolated from *n* = 6–12 biological replicates and assessed with Western blotting for protein expression of PDGFRα and PDGFRβ from dermal (**A**,**C**) and lung (**B**,**D**) fibroblasts. Relative expression was normalized with a total protein stain. Data represent the mean with SEM and each black dot represents a biological replicate. A one-way ANOVA was used to assess differences between conditions compared to control. * *p* < 0.05.

To assess feedback in the PDGF signalling pathway, we sought to investigate if the PDGF isoforms induce a negative feedback response through downregulation of the PDGFRα or PDGFRβ subunits in dermal and lung fibroblasts. In dermal fibroblasts, PDGFRα and PDGFRβ expression was not modulated by PDGF isoform stimulation versus the media control (Figure 9A, 9C). In lung fibroblasts, the expression of PDGFRα was unchanged following PDGF stimulation (Figure 9B). However, PDGF-AB and PDGF-CC stimulation resulted in significantly decreased PDGFRβ expression by lung fibroblasts compared to the media control (Figure 9D, *p* < 0.05). Downregulation of PDGFRβ by the PDGF isoforms may further explain why lung fibroblasts are not as responsive to PDGF signalling as dermal fibroblasts.

### 3.10. Dermal Fibroblasts Exhibit Elevated Baseline Phosphorylated ERK Expression

**Figure 10:**
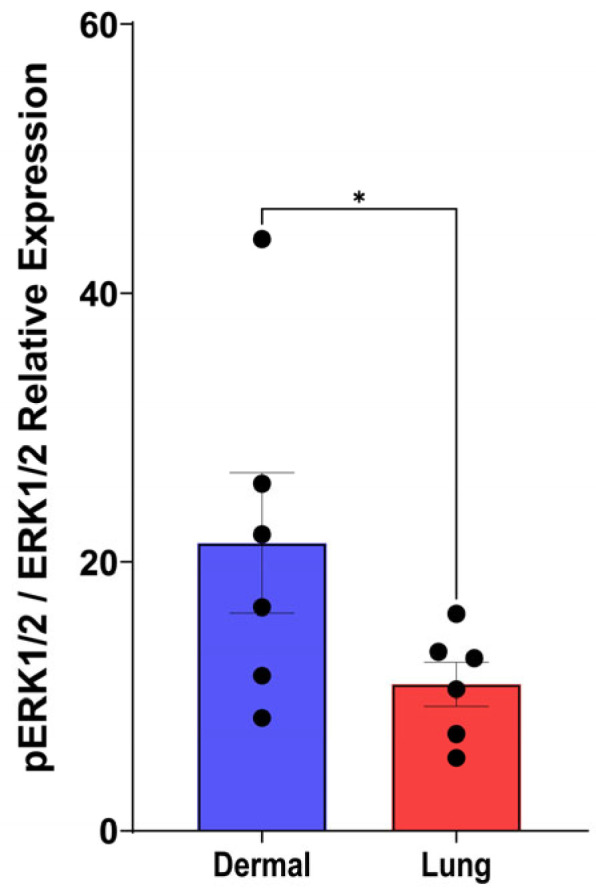
Baseline relative pERK1/2 expression is elevated in dermal fibroblasts. Fibroblasts were seeded at a density of 80,000 cells/well in a 6-well plate and were then serum-starved with DMEM containing 1% FBS upon reaching 80% confluency. Protein lysate was isolated from fibroblasts the following day and assessed with Western blotting for baseline protein expression of ERK1/2 and pERK1/2 from dermal and lung fibroblasts (*n* = 6 biological replicates). Expression was normalized with a total protein stain. Data represent the mean with SEM and each black dot represents a biological replicate. A *t*-test was used to assess differences in pERK1/2 as a fraction of total ERK1/2 expression between dermal and lung fibroblasts. * *p* < 0.05.

Given that PDGFRβ was the predominant receptor subunit expressed by dermal fibroblasts, we next examined potential differences in the downstream ERK1/2 (extracellular signal-regulated kinase) signalling pathway between dermal and lung fibroblasts. The ERK1/2 pathway is a well-established effector of PDGFRβ activation and can have downstream effects on proliferation, cytokine release, and ECM deposition in fibroblasts [30,31]. Under baseline conditions, dermal fibroblasts exhibited significantly higher levels of phosphorylated ERK1/2 (pERK1/2) compared to lung fibroblasts (Figure 10, *p* < 0.05). These elevated baseline levels of pERK1/2 in dermal fibroblasts correlate with their increased PDGFRβ expression and may explain their increased sensitivity to PDGF stimulation relative to lung fibroblasts.

## 4. Discussion

This study provides a comprehensive analysis of the effects of five PDGF isoforms on neonatal dermal and fetal lung fibroblast functions in relation to wound repair and fibrosis. Our data demonstrate that PDGF-BB is the predominant isoform driving fibroblast wound repair responses, including collagen I contraction, fibrotic IL-11 and IL-6 secretion, and proliferation. PDGF-AB and PDGF-CC also exerted isoform-specific effects, particularly on dermal fibroblast proliferation and collagen I contraction, whereas PDGF-AA had minimal effects on fibroblast functions. Notably, none of the PDGF isoforms induced myofibroblast differentiation or ECM deposition, highlighting that PDGF does not enhance all profibrotic fibroblast functions. In summary, these data indicate that PDGF isoforms induce distinct fibrotic fibroblast features with differential effects in the skin and lung, which underscores the potential for PDGF-BB-targeted inhibition strategies in fibrotic skin and lung diseases such as SSc and IPF.

In fibrosis, excessive ECM remodelling and contraction are hallmark features of dysregulated fibroblast function [32]. In the clinic, this is assessed in the skin using the modified Rodnan skin score (mRSS), a marker to assess skin thickness or hardness in patients [33]. *In vitro*, we used a 3D collagen I gel to model fibroblast contraction and identified that the PDGF isoforms AB, BB, CC, and DD significantly enhanced collagen contraction in both dermal and lung fibroblasts, with PDGF-BB having the most pronounced effect. Increased fibroblast contraction with PDGF signalling could contribute to ECM density in fibrotic tissues, increasing mechanical tension, and mechanotransduction [34]. 

Given the strong contractile induction of fibroblasts by PDGF, we sought to investigate the effect of PDGF-induced myofibroblast differentiation. We found no significant change in α-SMA expression indicative of myofibroblast differentiation following PDGF isoform stimulation in both dermal and lung fibroblasts. The absence of α-SMA upregulation suggests that PDGF isoforms may prime fibroblasts for ECM contraction without inducing differentiation to a myofibroblast phenotype. During the compaction of collagen gels, fibroblasts have been shown to remain elongated and use F-actin cytoplasmic stress fibres, without the requirement for the incorporation of α-SMA to form stress fibres [35]. In support of our findings, a previous study investigating the effect of PDGF-AB stimulation on α-SMA gene expression in primary adult dermal fibroblasts also found no α-SMA gene upregulation [7]. Further, in a study assessing cardiac fibroblasts, the authors found that basic fibroblast growth factor significantly increased fibroblast contraction of a collagen gel while simultaneously decreasing overall α-SMA and collagen I expression [36]. Together, these results support our data of enhanced dermal and lung fibroblast contractility in the absence of upregulated α-SMA expression or collagen I synthesis.

Proliferation is another hallmark of fibrotic fibroblast activation [15]. PDGF-AB, BB, and CC significantly enhanced dermal fibroblast proliferation, whereas lung fibroblasts were unresponsive to PDGF-induced proliferation, despite high basal proliferation. PDGF-AA, AB, and BB have previously been shown to induce proliferation in two other fetal lung fibroblast cell lines, IMR-90 and WI38, with a range of PDGF concentrations up to 20 ng/mL [37]. Additionally, primary adult lung fibroblasts treated with PDGF-BB have been shown to exhibit enhanced proliferation [37,38]. Our observed absence of a proliferative response in lung fibroblasts could reflect differences in the method of measurement, donor variation, growth factor concentration, or statistical analysis. It is important to note that in the current study we tested all five PDGF isoforms with rigorous correction for multiple comparisons, whereas previous studies did not. When our data were assessed using Fisher’s LSD test, not correcting for multiple comparisons, PDGF-AB, BB, and CC did significantly induce proliferation in fetal lung fibroblasts, similar to previous studies (Supplemental Figure S2). This finding indicates that the statistical approach used most likely reflects the differences in proliferation in lung fibroblasts between the three studies. Additionally, in response to TGF-β1 treatment, we also found differences in proliferation compared to the existing literature. Fibroblasts isolated from porcine dermal and murine cardiac tissue have previously been shown to decrease proliferation with TGF-β1 treatment, but in human and rat lung fibroblasts, TGF-β1 induced proliferation in serum-free conditions [39,40,41,42]. Thus, the differences seen with neonatal dermal and fetal lung fibroblasts in media supplemented with 1% serum may reflect differences in species, cell origin or cell culture conditions. Important next steps will be future experiments with adult-derived dermal and lung fibroblasts also isolated from fibrotic skin and lung diseases to assess the PDGF isoforms with rigorous statistical testing for multiple comparisons. In SSc, fibroblasts are known to resist apoptosis, enabling their persistence with elevated matrix protein and profibrotic cytokine expression [16,43]. It will be important to determine if inhibition of PDGF, especially PDGF-BB, slows the proliferation of fibroblasts in fibrotic skin conditions such as SSc.

PDGF-mediated cytokine secretion in dermal and lung fibroblasts was isoform and tissue-specific. PDGF-BB was the primary inducer of IL-11 and IL-6 in dermal fibroblasts, while multiple isoforms (AB, BB, CC, and DD) induced IL-11 in lung fibroblasts. IL-11 is a central mediator of fibrosis and central effector of fibrotic processes downstream from several molecules, including TGF-β1 [27,44]. In animal models of lung fibrosis, IL-11 plays a major role in mediating profibrotic processes such as myofibroblast differentiation, ECM deposition, epithelial-to-mesenchymal transition, and proinflammatory cytokine release [26]. Our results suggest that PDGF-BB may drive profibrotic signalling partly through IL-11, particularly in lung fibroblasts. Nintedanib, an orally administered multi-tyrosine kinase inhibitor with affinity for the PDGF receptor, attenuates lung fibrosis, but has limited effects on skin fibrosis in SSc, which may reflect tissue-specific effects of PDGF isoform signalling [45]. It is possible that the lack of therapeutic effect of nintedanib observed in the skin could be attributed to these tissue and isoform-specific effects of PDGF, such as limited isoform-induced IL-11 release from dermal fibroblasts, compared to lung fibroblasts.

In this study, PDGF-BB reduced collagen I deposition in dermal fibroblasts, and no PDGF isoform significantly modulated fibronectin expression in either cell type. However, within the literature, there have been several conflicting reports. PDGF-BB has been shown to induce the expression of both collagen I and fibronectin ECM proteins from primary human lung fibroblasts [30]. Similarly, in primary human dermal fibroblasts, a 40 ng/mL treatment of PDGF-BB significantly stimulated the expression of collagen I and fibronectin proteins [46]. Conversely, PDGF-AB-treated healthy dermal and lung fibroblasts downregulated the expression of the *COL1A1* gene when cultured with approximately 80 ng/mL of the cytokine [8]. In wound-isolated fibroblasts, PDGF-AA and BB isoforms induced dose-dependent inhibition of collagen I gene expression, with PDGF-AB stimulating collagen I gene expression at concentrations below 30 ng/mL [47]. Additionally, treatment with PDGF-AA, PDGF-AB, or PDGF-BB has been reported to have no effect on collagen synthesis in fetal lung fibroblasts [37]. The variable effects of PDGF on collagen I reported in the literature may reflect differences in fibroblast origin, PDGF isoform concentration, and the context of signalling. The comprehensive assessment of all PDGF isoforms on both dermal and lung fibroblasts in this study helps shed light on PDGF isoform differences. An important next comparison will be how the PDGF isoforms affect adult fibroblasts derived from the skin and lung of patients with fibrotic diseases. 

Collectively, our data demonstrate clear isoform-specific and tissue-specific differences in fibroblast responses to PDGF. We therefore sought to explore the constituents of the PDGF signalling pathway, particularly the two receptor chains, PDGFRα and PDGFRβ that comprise the PDGF receptor complex. Dermal fibroblasts, with higher PDGFRβ expression, were more responsive to PDGF-BB, exhibiting increased contraction, proliferation, and proinflammatory cytokine release, while lung fibroblasts responded predominantly through IL-11 secretion and contraction. Further, dermal fibroblasts exhibited higher basal ERK1/2 phosphorylation, the major downstream signalling cascade for the PDGF receptor, indicating that the elevated receptor level leads to increased ERK1/2 signalling. These findings provide a mechanistic basis for the heightened PDGF responsiveness in dermal fibroblasts compared to lung fibroblasts.

It is understood that PDGF-BB binds to all three PDGF receptor dimer complexes with high affinity [17]. In dermal fibroblasts, higher PDGFRβ expression may increase the likelihood of PDGF-BB binding to PDGFRββ receptor complexes and initiating intracellular signalling. These findings underscore the importance of considering fibroblast heterogeneity and receptor expression profiles when designing PDGF-targeted therapies for fibrotic diseases.

The limitations of this study include the use of neonatal dermal and fetal lung fibroblast cell lines, which may not fully recapitulate the differential effects of PDGF isoform signalling in adult fibroblasts. However, these human-derived, non-transformed, and non-cancer-derived neonatal dermal and fetal fibroblast cell lines are commercially available, enabling other researchers to reproduce these findings. These cell lines also allow for the growth of large numbers of cells, enabling biological replicates and comparison of many fibroblast functional assays in response to the multiple PDGF isoforms. Future experiments in adult dermal and lung fibroblasts will be important for assessing the expression profiles of the PDGFRα and PDGFRβ receptor chains. Additionally, our experiments were conducted in healthy fibroblasts, which may differ in signalling responses compared to fibroblasts derived from fibrotic lesions. Despite these limitations, our study provides a detailed isoform-specific map of the effects of PDGF on fibroblast wound repair and fibrotic functions, offering mechanistic insight into the role of PDGF signalling in fibrosis.

In conclusion, this comprehensive study of the PDGF family demonstrates that PDGF-BB is the predominant isoform driving fibroblast proliferation, contractility, and IL-11-mediated profibrotic signalling, but not ECM synthesis and myofibroblast differentiation. The differential responses between dermal and lung fibroblasts highlight the potential for tissue-targeted, isoform-specific therapeutic strategies in diseases, such as SSc or IPF, which may provide more precise modulation of fibroblast activity than systemic pan-PDGF inhibition.

## Data Availability

The original contributions presented in this study are included in the article/Supplementary Materials. Further inquiries can be directed to the corresponding author.

## Supplementary Materials

The following supporting information can be downloaded at: https://www.mdpi.com/article/10.3390/cells15070637/s1, Figure S1. Dose response of PDGF isoforms. Figure S2. PDGF-AB, BB, and CC enhance lung fibroblast proliferation. Figure S3. PDGF isoform stimulation does not induce cell death.

## Abbreviations

The following abbreviations are used in this manuscript:

3D	3-dimensional
α-SMA	α-smooth muscle actin
ANOVA	Analysis of variance
BSA	Bovine serum albumin
DMEM	Dulbecco’s modified eagle medium
ECM	Extracellular matrix
ELISA	Enzyme-linked immunosorbent assay
ERK	Extracellular signal-regulated kinase
FBS	Fetal bovine serum
IL	Interleukin
ILD	Interstitial lung disease
IPF	Idiopathic pulmonary fibrosis
LDH	Lactate dehydrogenase
LSD	Least significant difference
PBS	Phosphate-buffered saline
PSF	Penicillin-streptomycin fungizone
PDGF	Platelet-derived growth factor
PDGFR	Platelet-derived growth factor receptor
SSc	Systemic sclerosis
SEM	Standard error of the mean
TGF-β	Transforming growth factor-β
