# Supplementary material for: Tissue and Isoform-Specific Effects of Platelet-Derived Growth Factor on Neonatal-Derived Dermal and Fetal-Derived Lung Fibroblast Profibrotic Functions
Source: Cells. 2026 Apr 1;15(7):637. doi: 10.3390/cells15070637 (PMC13072191; doi:10.3390/cells15070637)
Supplement: Supplementary file 1 [file cells-15-00637-s001.zip › cells-4200107-supplementary.pdf]

SUPPLEMENTARY MATERIALS

Tissue and Isoform-Specific Effects of Platelet-Derived Growth Factor on Neonatal-Derived Dermal and Fetal-Derived Lung Fibroblast Profibrotic Functions

Brandon Kohlen[1,2,*], Raveen Badyal[1,2], Kevin J. Keen[1,3,4], James V. Dunne[1,4] and Tillie-Louise Hackett[1,2]

1 Centre for Heart Lung Innovation, St. Paul's Hospital, Vancouver, BC V6Z 1Y6, Canada

2 Department of Anesthesiology, Pharmacology and Therapeutics, The University of British Columbia, Vancouver, BC V6T 1Z3, Canada

3 Department of Mathematics and Statistics, The University of Northern British Columbia, Prince George, BC V2N 4Z9, Canada

4 Department of Medicine, The University of British Columbia, Vancouver, BC V6T 1Z3, Canada

* Correspondence: brandon.kohlen@hli.ubc.ca

Supplemental Results

Supplemental Figure S1. Dose response of PDGF isoforms.

In dermal and lung fibroblasts, treatment with PDGF-AB, PDGF-BB, PDGF-CC or PDGF-DD induced a dose-dependent increase in the release interleukin (IL)-11 (Supplemental Figure S1). A dose-dependent response was not observed following PDGF-AA stimulation in both dermal and lung fibroblasts.

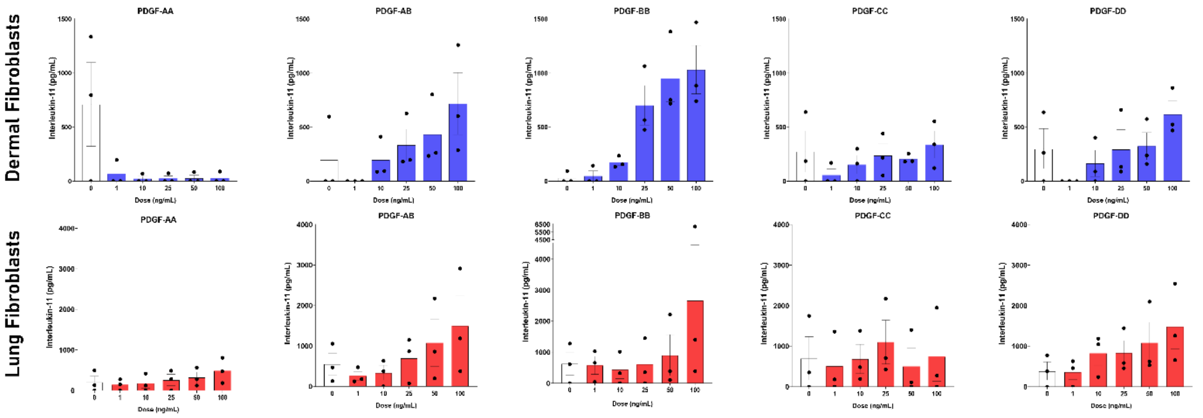

Supplemental Figure S1. Dose-dependent interleukin (IL)-11 response of PDGF-treated dermal and lung fibroblasts. Dermal and lung fibroblasts were seeded at a density of 80,000 cells/well in a 6-well plate, stimulated with 1, 10, 25, 50, and 100 ng/mL PDGF-AA, AB, BB, CC, or DD for 72 hours, then assessed for IL-11 release using ELISA. Data represent the mean with SEM and each black dot represents a biological replicate.

Supplemental Figure S2: PDGF-AB, BB, and CC enhance lung fibroblast proliferation.

PDGF-AB, PDGF-BB, and PDGF-CC significantly stimulated an increase in the fold-change of lung fibroblast proliferation, as assessed by Fisher's LSD test (Supplemental Figure S2, $p < 0.05$).

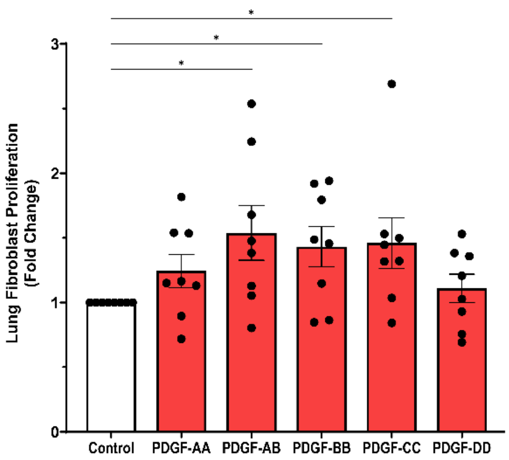

Supplemental Figure S2. PDGF-AB, BB, and CC enhance lung fibroblast proliferation. Lung fibroblasts were seeded at a density of 80,000 cells/well in a 6-well plate and stimulated with 25 ng/mL PDGF-AA, AB, BB, CC, or DD. Data is shown as the fold-change in cell number. Data represent the mean with SEM and each black dot represents a biological replicate. Fisher's LSD test was used to assess differences between groups compared to control. * $p < 0.05$.

Supplemental Figure S3: PDGF isoform stimulation does not induce cell death.

The effect of PDGF isoform stimulation on cell death was assessed with a lactate dehydrogenase (LDH) cytotoxicity assay (Supplemental Figure S3). In both dermal and lung fibroblasts, all PDGF isoforms exhibited LDH release comparable to cells treated with control media and were below the threshold of the positive control.

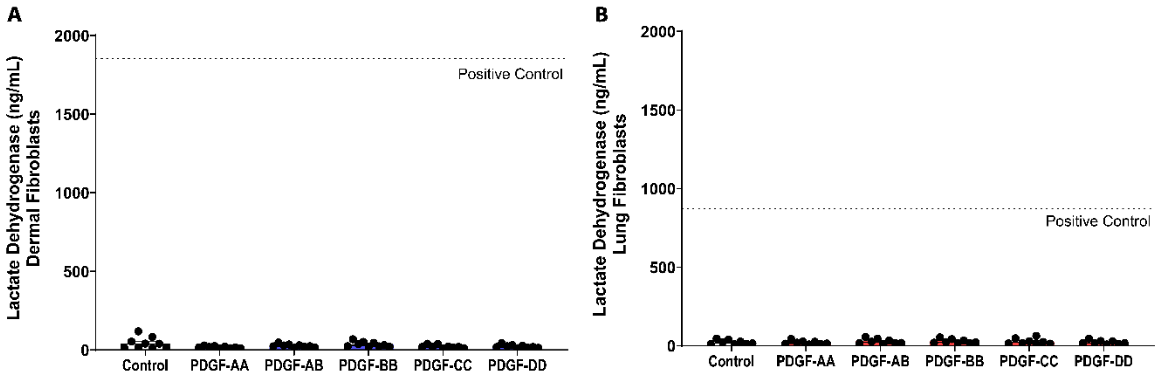

Supplemental Figure S3. PDGF isoform stimulation does not induce cell death in dermal or lung fibroblasts. Fibroblasts were seeded at a density of 80,000 cells/well in a 6-well plate, stimulated with 25 ng/mL PDGF-AA, AB, BB, CC, or DD for 72 hours, then assessed for lactate dehydrogenase (LDH) release using the LDH assay kit (cat. ab65393, Abcam) and a standard curve of recombinant human lactate dehydrogenase protein (cat. ab93699, Abcam), as shown for dermal (A) and lung (B) fibroblasts. Data represent the mean with SEM and each black dot represents a biological replicate. The "Positive Control" represents the amount of LDH release from the lysis of 0.5 million dermal or lung fibroblasts.
